# Time Domain Strain/Stress Reconstruction Based on Empirical Mode Decomposition: Numerical Study and Experimental Validation

**DOI:** 10.3390/s16081290

**Published:** 2016-08-16

**Authors:** Jingjing He, Yibin Zhou, Xuefei Guan, Wei Zhang, Weifang Zhang, Yongming Liu

**Affiliations:** 1 School of Reliability and System Engineering, Beihang University, Beijing 100191, China; zhangwei.dse@buaa.edu.cn (W.Z.); zhangweifang@buaa.edu.cn (W.Z.); 2 School of Energy and Power Engineering, Beihang University, Beijing 100191, China; zhouyibin@buaa.edu.cn; 3 Siemens Corporation, Corporate Technology, 755 College Rd. E., Princeton, NJ 08540, USA; xf.guan@gmail.com; 4 School for Engineering of Matter, Transport and Energy, Arizona State University, Tempe, AZ 85281, USA; yongming.liu@gmail.com

**Keywords:** limited sensor data, structural health monitoring, strain/stress response reconstruction, empirical mode decomposition

## Abstract

Structural health monitoring has been studied by a number of researchers as well as various industries to keep up with the increasing demand for preventive maintenance routines. This work presents a novel method for reconstruct prompt, informed strain/stress responses at the hot spots of the structures based on strain measurements at remote locations. The structural responses measured from usage monitoring system at available locations are decomposed into modal responses using empirical mode decomposition. Transformation equations based on finite element modeling are derived to extrapolate the modal responses from the measured locations to critical locations where direct sensor measurements are not available. Then, two numerical examples (a two-span beam and a 19956-degree of freedom simplified airfoil) are used to demonstrate the overall reconstruction method. Finally, the present work investigates the effectiveness and accuracy of the method through a set of experiments conducted on an aluminium alloy cantilever beam commonly used in air vehicle and spacecraft. The experiments collect the vibration strain signals of the beam via optical fiber sensors. Reconstruction results are compared with theoretical solutions and a detailed error analysis is also provided.

## 1. Introduction

Structural health monitoring (SHM) is one of the key components in engineering systems, civil infrastructures, and smart structures [1,2,3,4]. Due to the increasing complexity and high reliability demand of those systems, damage and system performance degradation need to be quantified accurately for maintenance purposes [5,6]. To monitor the status of a target system, measurements of system condition variables are taken either by sensors installed on the system or field non-destructive inspections [7,8,9]. In particular, using smart sensors for structural health monitoring has drawn a lot of attention in the SHM community due to flexibility of smart sensors and advances in composite materials [9,10,11,12]. A commonly used classification system for damage identification methods defines four levels of damage identification: (1) determine the existence of the damage in a structure; (2) identify the location of the damage; (3) quantify the severity of the damage; and (4) estimate the remaining life of the system [13]. The difficulty of having a reliable result increases as the system complexity increases. Therefore, obtaining an accurate estimation of the remaining useful life of a damaged system is a practical challenge. For most mechanical systems subjected to fatigue loads, fatigue crack propagation is one of major failure mechanisms. Fatigue damage is highly dependent on the applied stress history under realistic service conditions. The practical challenge for fatigue life prediction in such cases is that the stress and fatigue load in the vicinity of the fatigue crack is very difficult to infer. Direct measurement of the stress or strain in the damaged location is very difficult because the damage location is not known a priori [14]. Furthermore, it is highly nontrivial to place additional sensors at the damaged spot because the local geometry of the damage spot may be too complex to install a new sensor and the service condition of the system may not allow one to install new sensors. Using measurements from pre-installed sensors to infer the information about the critical spot, i.e., a fatigue crack damaged location without direct sensor measurements, may be the only practical option in realistic situations. Therefore, extrapolation to a critical spot from measurements located in a remote spot is a necessary step for fatigue prognostics and the remaining life prediction. Recent advances of dynamical response extrapolation include frequency-domain methods and direct time-domain methods. Several studies suggest using the concept of transmissibility [15,16,17]. The basic idea is to define a transformation function between the measurement spot and the desired spot. The method requires a minimal number of locations to obtain the dynamical responses of the desired spot. Direct time domain reconstruction of dynamical responses for acceleration and velocity using empirical mode decomposition (EMD) method is demonstrated in [14]. The basic idea of EMD-based time-domain reconstruction method is to decompose the measurement data using EMD approach [18] with the intermittency criteria. The extrapolation function from a measurement spot to a desired spot is based on the mode shape ratio of the two positions. EMD has been widely used in structural health monitoring, such as for systems with joints [19], non-smooth contacts [20,21] and rotating machinery [22]. EMD has its own limitations in distinguishing different components in narrowband signals. Various improvements have been made to EMD to address these limitations, such as implementation of masking signals [23], ensemble EMD [24], step-by-step EMD [25,26] and heterodyning [27]. However, these methods cannot be directly applied to obtain the strain and stress responses for structural health monitoring, and it has not been validated by real field sensor measurements.

The objective of this study is to develop a direct time domain strain/stress reconstruction method using sparse and remote sensor measurements. The proposed method extends the previous EMD-based time-domain reconstruction method by using the finite element model to derive a strain transformation function in modal coordinates. EMD method with intermittency criteria is employed in the proposed method to decouple the remote strain measurements into modal coordinates, and the extrapolation of the strain and stress responses is made using the strain and stress transformation function. The overall method is demonstrated using a two-span beam-like structure. A more complex 19956-DOF airfoil structure is used to further investigate the effectiveness and performance of the method under different signal-to-noise ratios. Besides, the presented method has been experimentally investigated on an aluminium alloy cantilever beam. The beam was excited with an impulse hammer at the free end and its vibration signals were captured by optical fiber sensors bonded to the upper surface of the beam.

The paper is organized as follows: first, the EMD method for signal decomposition is briefly introduced, and the required signal filtering process using the intermittency criteria is discussed. Next, the transformation equation for strain and stress is derived using the FE model of the structure. Following this, a numerical two-span beam-like structure and a simplified airfoil structure are used to demonstrate and validate the proposed method. A set of experiments were conducted on an aluminium alloy cantilever beam to investigate the effectiveness of the method. Finally, several conclusions are drawn based on the current study.

## 2. Strain and Stress Reconstruction Methodology

**Figure 1:**
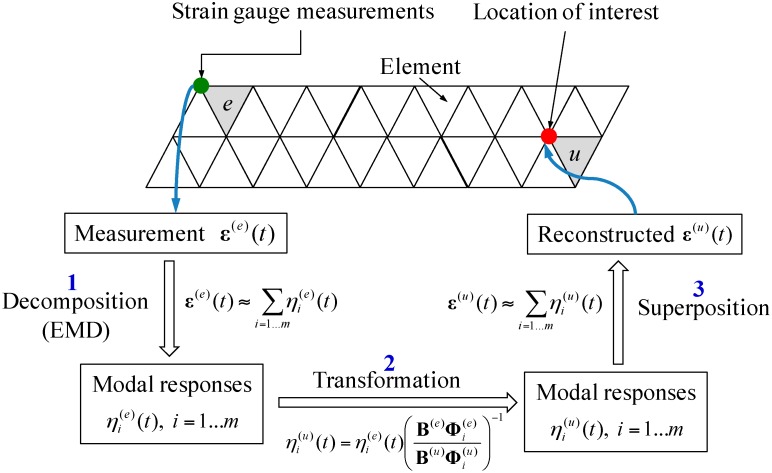
Flowchart of the overall strain reconstruction procedure using remote strain measurements.

The proposed strain and stress reconstruction method is based on three pieces of information: The measurement data from one or multiple strain sensors, the structural model, and locations of sensors and hot spots. The overall process for the reconstruction involves several steps, as illustrated in Figure 1. Details of each step are discussed in this section.

### 2.1. Extraction of Modal Responses from Measurement Data Using EMD Method

The first step of the reconstruction method is to decompose the measured time domain signals from strain sensors into a set of responses in modal coordinates. This step can be completed using empirical mode decomposition (EMD) method with intermittency criteria. For the completeness of the paper, the EMD method with intermittency criteria is briefly introduced.

The basic idea of EMD method is to break down the original signal into a set of intrinsic mode functions (IMF) and a residual term. An IMF is a function that has a mean value of zero and only one extreme between zero crossings. The IMFs form a complete and nearly orthogonal basis for the original signal, allows for varying frequencies in time to be preserved, which is hidden in the Fourier domain or in wavelet coefficients [18]. For a given time series data *y*(*t*), the EMD method uses the sifting process to obtain IMFs [18]. The original signal *y*(*t*) can be expressed as the summation of n IMFs and a residual term, as shown in Equation (1):

(1) $$y(t)=\sum_{i=1}^{n}f_{i}(t)+r(t)$$

where *f_i_*(*t*) is the *i*th IMF and *r*(*t*) is the residue. The term r(t) also represents the mean trend or constant for this signal [28].

Each of the IMFs obtained from the above standard sifting process may contain several frequency components, and is not a good approximation to the modal responses. To ensure each of the IMFs contains only one frequency component (i.e., the modal responses corresponding to some natural frequency), an intermittency frequency denoted by *ω_int_* must be imposed in the sifting process. The idea is to remove all frequency components lower and larger than *ω_int_*, and this can be done prior to or within the sifting process using a band-pass filter. The process to obtain the modal response corresponding to the *i*th natural frequency *ω_i_* is discussed in [28]. These IMFs have several characteristics: (1) Each IMF contains the intrinsic characteristics of the signal; (2) Once an IMF is obtained, the next IMF will not have the same frequency at the same time instant [29,30]; and (3) The first IMF for each IMFs series is considered to be the approximation of modal response. Using the sifting process with intermittency criteria, the original signal expression can be written as Equation (2):

(2) $$y(t)\approx \sum_{i=1}^{m}x_{i}(t)+\sum_{i=1}^{n-m}f_{i}(t)+r(t)$$

where *x_i_*(*t*) is the modal response (that is also an IMF) for the *i*th mode. Terms *f_i_*(*t*) (*i* = 1,2,…,*n–m*) are other IMFs but not modal responses.

### 2.2. Transformation Equations for Strain and Stress Responses

To reconstruct the strain and stress responses at a location (without direct measurements) using strain measurement data from a remote location, transformation equations are needed to establish the physical relationship between the two locations. For a general structure, finite element model (FEM) can be used as the structural model to derive transformation equations. Considering a general FEM to describe the structure under analysis, the system dynamics equation can be expressed as:

(3) $$M\ddot{X}+C\dot{X}+KX=F$$

where **M**, **K** and **C** are the mass, stiffness, and damping matrices, respectively. **X** is the displacement vector and **F** is the load vector. For practical structures subject to stochastic excitations, **F** is unknown and directly solving Equation (3) to obtain the dynamical responses of a sensor inaccessible location is not possible. However, the finite element method allows for correlating displacement responses of two different DOFs in the modal coordinates through the mode shape matrix. The mode shape matrix can readily be obtained by solving the eigenvalue problem of:

(4) $$\left[\Phi ,\lambda ]=\text{eig([}M^{-1}K]\right)$$

where Φ and λ are the eigenvectors and eigenvalues, respectively. Φ (Equation (5)) is also referred to as the mode shape matrix. λ corresponds to the natural frequencies of the structure, i.e., $\lambda ={\left(2\pi f\right)}^{2}$ where **f** is the vector of natural frequencies:

(5) $$\Phi =\left[\begin{array}{ccc} \varphi_{11} & \cdots & \varphi_{n1}\\ \vdots & \ddots & \vdots \\ \varphi_{1n} & \cdots & \varphi_{nn} \end{array}\right]$$

The physical meaning of Φ can be interpreted as follows: each column of Φ represents a mode and each component in the column represents the displacement contribution of a DOF in the structure. For example, *ϕ_ij_* represents the displacement contribution from DOF *j* under mode *i*. Since the model shape matrix is a constant once the number of the DOF and the discretization topology of the structure are determined, the ratio of displacement contribution of one DOF to that of another DOF is also a constant. This characteristic indicates that the responses of one DOF under modal coordinates allows for the calculation of responses of another DOF under modal coordinates. Denote the responses under modal coordinates as *δ_ij_*, where *i* and *j* represent the mode index and the DOF index, respectively, the physical meaning of the modal response relationship between two DOFs can be expressed as:

(6) $$\frac{\varphi_{ie}}{\varphi_{iu}}=\frac{\delta_{ie}}{\delta_{iu}}$$

where the subscript *e* represents the DOF (location) which physical responses can be measured by sensors and *u* represents the DOF that is inaccessible for sensor measurements. *δ_ij_*(*t*) corresponds to the modal responses components for the overall physical displacement responses of **X***_j_*(*t*) for DOF *j* at a time index *t*. If the physical displacement responses of the DOF e having sensor measurements can be decomposed into its modal responses, i.e., $X_{e}\left(t\right)\approx \underset{i=1\ldots m}{\sum }\delta_{ie}\left(t\right)$, using Equation (6), the physical displacement responses of the sensor inaccessible DOF *u* can be obtained as:

(7) $$X_{u}(t)\approx \sum_{i=1...m}\left[\delta_{ie}(t){\left(\frac{\varphi_{ie}}{\varphi_{iu}}\right)}^{-1}\right]$$

where i=1...m denotes the participating modes. It should be noted that Equation (7) holds for displacement X(t), velocity $\dot{X}(t)$, and acceleration $\ddot{X}(t)$, but it cannot be directly applied to reconstructions for strain and stress responses. Since Equation (6) holds true for any arbitrary two DOFs, a more general equation can be obtained for any given time index *t*:

(8) $$\Phi_{i}=\alpha \delta_{i}$$

where $\Phi_{i}$ is the *i*th column vector in the mode shape matrix Φ, $\delta_{i}$ is the *i*th modal responses for all DOFs, α is a scalar constant for a given time index *t*.

Denote the strain and stress responses for an element (in the FE model) indexed by *k* at time index *t* as **ε**^(*k*)^ and **σ**^(*k*)^. From the finite element formulation, the strain and displacement has the following relationship:

(9) $$\varepsilon^{\left(k\right)}=B^{\left(k\right)}\cdot X^{\left(k\right)}$$

where **B**^(*k*)^ is the strain-displacement matrix for element *k* and **X**^(*k*)^ is the displacement response vector consisting of all DOFs of element *k*. The expression of **B**^(*k*)^ usually has the form:

(10) $$B^{\left(k\right)}=LN^{\left(k\right)}$$

where **L** is the differential operator and **N**^(*k*)^ is the matrix of shape functions for element *k*. Using Equation (8), the following equation is obtained under modal coordinates:

(11) $$B^{\left(k\right)}\Phi_{i}^{\left(k\right)}=\alpha B^{\left(k\right)}\delta_{i}^{\left(k\right)}$$

The term $B_{k}\delta_{i}^{\left(k\right)}$ (for simplicity, denoted as $\eta_{i}^{\left(k\right)}$) is the strain response vector associated with *i*th mode and *k*th element under modal coordinates. The transformation equation for strain responses under *i*th mode between two elements indexed by *e* and *u* can be obtained by using Equation (11) as:

(12) $$\frac{B^{\left(e\right)}\Phi_{i}^{\left(e\right)}}{B^{\left(u\right)}\Phi_{i}^{\left(u\right)}}=\frac{\alpha B^{\left(e\right)}\delta_{i}^{\left(e\right)}}{\alpha B^{\left(u\right)}\delta_{i}^{\left(u\right)}}=\frac{\eta_{i}^{\left(e\right)}}{\eta_{i}^{\left(u\right)}}$$

The result of Equation (12) indicates that if the physical strain responses measured at one location (represented by element *e* in the FE model) can be decomposed to its modal responses, i.e., $\varepsilon^{\left(e\right)}\approx \underset{i=1\ldots m}{\sum }{\eta_{i}}^{\left(e\right)}$, the physical strain responses at a sensor inaccessible location (represented by element *u* in the FE model) can be reconstructed using the following transformation equation:

(13) $$\varepsilon^{\left(u\right)}(t)\approx \sum_{i=1...m}\left[\eta_{i}^{\left(e\right)}(t){\left(\frac{B^{\left(e\right)}\Phi_{i}^{\left(e\right)}}{B^{\left(u\right)}\Phi_{i}^{\left(u\right)}}\right)}^{-1}\right]$$

where i=1...m denotes the participating modes and all other notations are defined as before. The proposed method can be used to reconstruct the strain/stress in directions where sensor measurements are available. From mathematical point of view, the strain in a given direction (such as $\varepsilon_{xx}$, $\varepsilon_{xy}$, $\varepsilon_{yy}$……etc.) should have no difference in the overall procedure. Once the physical strain responses are reconstructed using Equation (13), the stress responses can be readily calculated using the following constitutive equation:

(14) σ(t)=cε(t)

where **c** is the material matrix. The constitutive equation for isotropic materials can be written explicitly as:

(15) $$\left\{\begin{array}{c} \sigma_{xx}\\ \sigma_{yy}\\ \sigma_{zz}\\ \sigma_{yz}\\ \sigma_{xz}\\ \sigma_{xy} \end{array}\right\}=\left[\begin{array}{cccccc} c_{11} & c_{12} & c_{12} & 0 & 0 & 0\\ & c_{11} & c_{12} & 0 & 0 & 0\\ & & c_{11} & 0 & 0 & 0\\ & & & (c_{11}-c_{12})/2 & 0 & 0\\ & sym. & & & (c_{11}-c_{12})/2 & 0\\ & & & & & (c_{11}-c_{12})/2 \end{array}\right]\left\{\begin{array}{c} \varepsilon_{xx}\\ \varepsilon_{yy}\\ \varepsilon_{zz}\\ \varepsilon_{yz}\\ \varepsilon_{xz}\\ \varepsilon_{xy} \end{array}\right\}$$

where $c_{11}=\frac{E(1-\nu )}{\left(1-2\nu )(1+v\right)}$, $c_{12}=\frac{E\nu }{\left(1-2\nu )(1+v\right)}$, and $(c_{11}-c_{12})/2=G$. Terms *E*, *v*, and *G* are Young’s modulus, Poisson’s ratio, and the shear modulus of the material, respectively. The relationship between the three material constants is:

(16) $$G=\frac{E}{2(1+\nu )}$$

Based on above discussions and derivations, the overall procedure for strain responses reconstruction is summarized in Figure 1.

A novel method for reconstructing strain/stress responses at the hot spots of the structures based on strain measurements at remote locations is presented in this work. Once the strain and stress responses of locations of interest are obtained using the above described method, the stress responses information can be used in the remaining useful life prediction, structural health monitoring and decision-making et al. Two numerical examples and a set of experiments are presented in the next section. The strain responses in the following numerical and experimental examples refer to strains in a single direction.

## 3. Numerical Examples

Two numerical examples are presented here. The first example is a two-span beam structure subject to random forces. The second example is a complex airfoil structure with 19,956 DOFs.

### 3.1. Example 1: A Numerical Beam Structure Example

**Figure 2:**
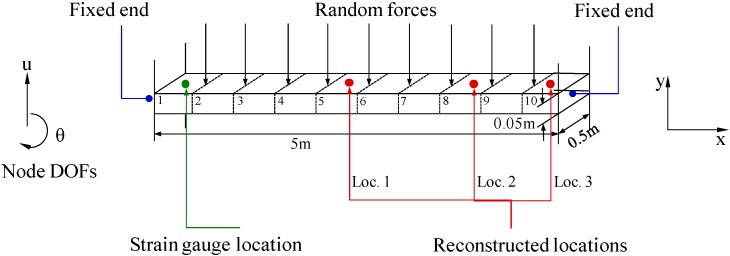
FE diagram of a beam structure with both ends fixed and applied forces. Synthesized strain measurement locations and the three reconstructed locations are the geometry centers of the surfaces of elements 1, 5, 8, and 10, respectively.

A beam structure is used to demonstrate the overall reconstruction methodology and to investigate the performance of the proposed method under different measurement noises. The beam is 5 m long, 0.5 m wide and 0.05 m thick. The Young’s modulus is 69,600 MPa and the density is 2730 kg/m^3^. The beam structure is divided into 10 equal segments in the FE model, as shown in Figure 2. Random forces are applied at all vertical direction DOFs of the FE model. The random forces are modeled as Gaussian white noise processes passed through a sixth order low-pass Butterworth filter with a 100 Hz cutoff. One percent of modal damping is considered. Displacement responses are calculated by solving the equation of motion of the beam based on its finite element model using mode superposition method. The sampling frequency is 1000 Hz. Strain responses are calculated using the strain-displacement matrix and Equation (9). The beam is modeled using Euler–Bernoulli beam theory and the 1 × 4 strain-displacement matrix is given by:

(17) $$B^{\left(k\right)}=-\frac{y^{\left(k\right)}}{{\left[L^{\left(k\right)}\right]}^{2}}\cdot \left[\begin{array}{cccc} 6\left(-1+\frac{2x^{\left(k\right)}}{L^{\left(k\right)}}\right) & 2L^{\left(k\right)}\left(-2+\frac{3x^{\left(k\right)}}{L^{\left(k\right)}}\right) & 6\left(1-\frac{2x^{\left(k\right)}}{L^{\left(k\right)}}\right) & 2L^{\left(k\right)}\left(-1+\frac{3x^{\left(k\right)}}{L^{\left(k\right)}}\right) \end{array}\right]$$

where the superscript (*k*) denotes the *k*th element, $L^{\left(k\right)}$ is the length of the element, $x^{\left(k\right)}$ and $y^{\left(k\right)}$ are the horizontal and vertical locations (within the element dimension) that the strain is computed, respectively. The displacement responses at a given time index *t*, $X^{\left(k\right)}(t)$, is a 4 × 1 matrix:

(18) $$X^{\left(k\right)}(t)=\left[\begin{array}{cccc} u_{1}^{\left(k\right)}(t) & \theta_{1}^{\left(k\right)}(t) & u_{2}^{\left(k\right)}(t) & \theta_{2}^{\left(k\right)}(t) \end{array}\right]$$

where $u_{1}^{\left(k\right)}(t)$, $\theta_{1}^{\left(k\right)}(t)$, $u_{2}^{\left(k\right)}(t)$ and $\theta_{2}^{\left(k\right)}(t)$ are the vertical and rotational displacements of the four DOFs of the element. The result of $\varepsilon^{\left(k\right)}(t)=B^{\left(k\right)}X^{\left(k\right)}(t)$ is the x-direction strain of the location specified by ($x^{\left(k\right)}$,$y^{\left(k\right)}$) in the element. After the strain response generations, noise signals are added to each point in the strain responses to represent the noisy sensor measurements. The noise signals are Gaussian pulse process with root mean square (RMS) setting to a percentage of the largest RMS of the strain responses. The noise terms are specified using the noise level in this study, for example, a 10% noise level refers to the noise elements are Gaussian pulse processes with RMS 10% of the largest RMS of the strain responses.

**Figure 3:**
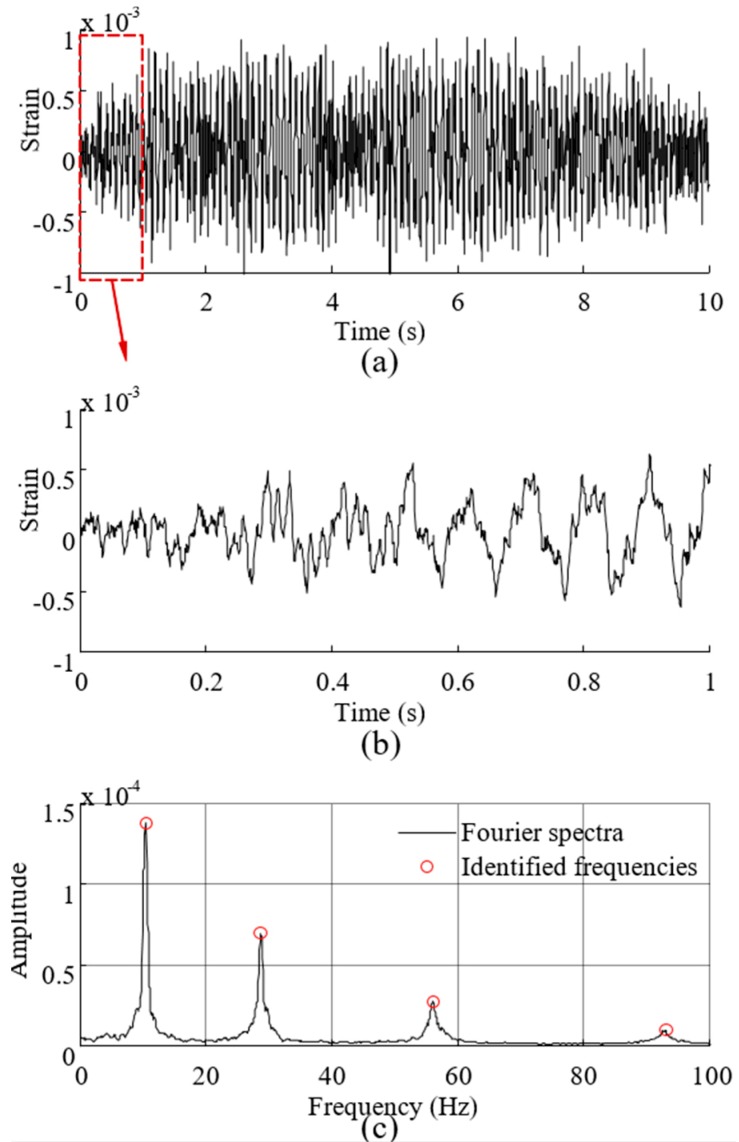
The synthesized strain measurement data and the Fourier spectra of the data. (**a**) Entire 10 s measurement data; (**b**) Measurement data (0–1 s); and (**c**) Fourier spectra of the measurement data.

**Figure 4:**
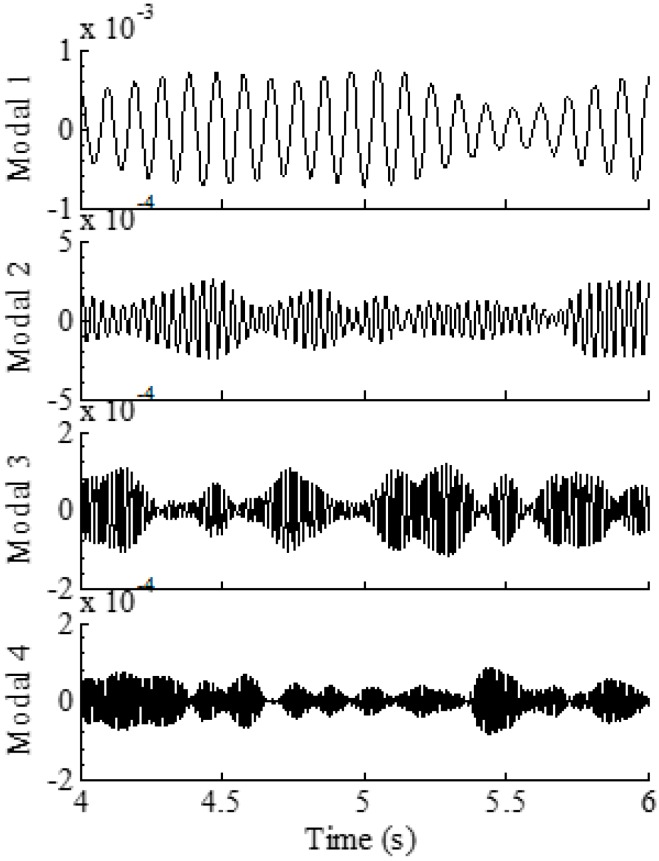
Four modal responses of the strain measurement data obtained using EMD method with intermittency criteria.

To demonstrate the overall reconstruction methodology, 10-s noisy (10% RMS) sensor measurement data are generated using the above described procedure. The location of the assumed sensor is at (0.25 m, 0.05 m) of the element close to the left fixed end (labeled 1 in Figure 2). The synthesized strain measurement data are shown in Figure 3a (the 0–1 s data are shown in Figure 3b for clear demonstration) and the Fourier spectra of the data is shown in Figure 3c, where four frequencies can be easily identified. The identified frequencies from the Fourier spectra are (10.38 Hz, 28.69 Hz, 56.22 Hz, 93.15 Hz), and they are used to design the band-pass filters. Frequency ranges for each band-pass filter are given in Table 1. EMD method with intermittency criteria is employed first to obtain modal responses corresponding to the four natural frequencies from the measurement data. Figure 4 presents the results of modal responses of the strain measurement data in Figure 3a. For clear demonstration, the middle portion (4–6 s) data are shown.

**Figure 5:**
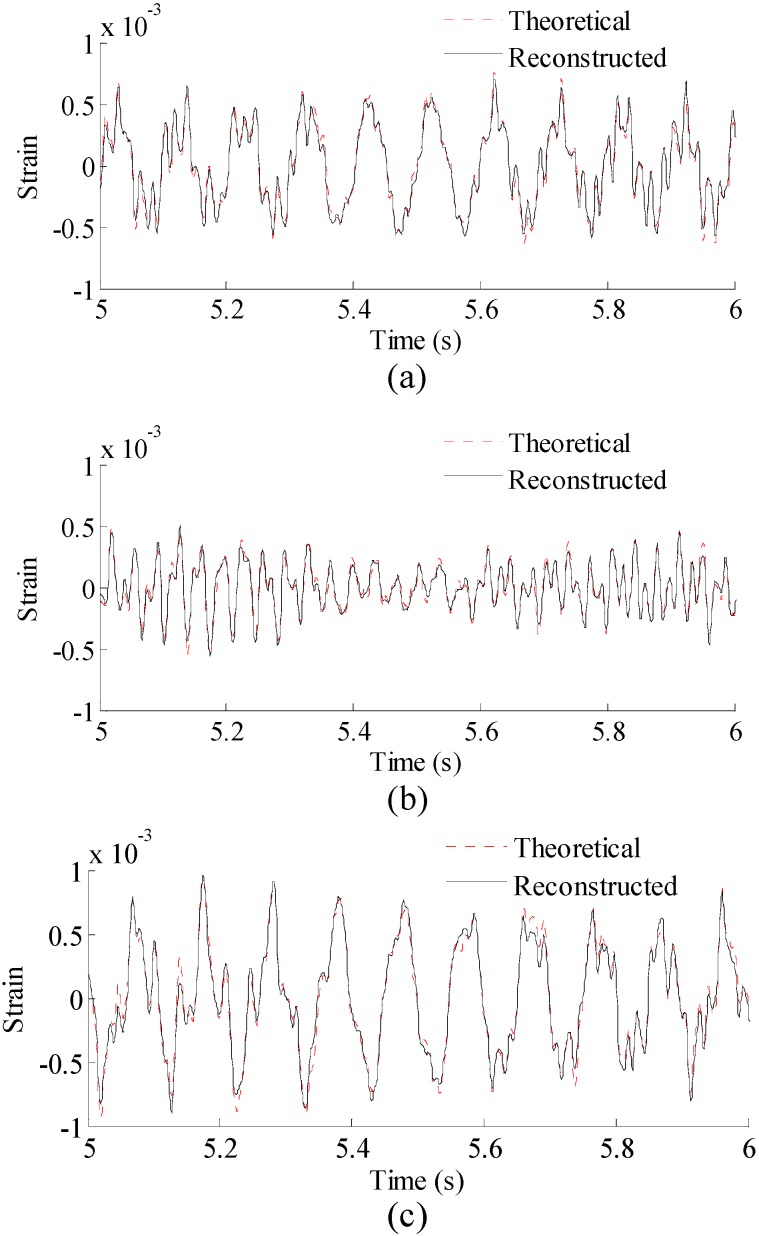
Reconstructed and theoretical strain responses for three locations (Loc. 1–3, Loc. in the paper are short form of location number) in Figure 2. Results are concentrated on 5–6 s for clear presentation. (**a**) Results for Loc. 1; (**b**) results for Loc. 2; and (**c**) results for Loc. 3.

The four modal response results are used in Equation (13) to obtain the strain responses for the three locations of interest shown in Figure 2. The 1 × 4 matrix $B_{k}=\left[\begin{array}{cccc} 0 & 0.05 & 0 & -0.05 \end{array}\right]$ can be used for all elements (i.e., *k* = 1…10) due to the uniform discretization of the beam. The method can also be used to efficiently map the strains over the entire structure, if no specific locations are chosen. This examples are executed to reconstruct the strains over the entire beam (the rest 9 elements) using the strain signal of the element 1. The reconstructed results of the rest 9 elements agree well with the theoretical results. For clear presentation here, three typical locations of interest (elements 5, 8, 10) are chosen to be the locations of interest in this example, and the results are shown in Figure 5. It can be observed that the reconstructed results are very close to the theoretical results for the three locations, considering the measurement data with 10% RMS measurement noises. 

**Figure 6:**
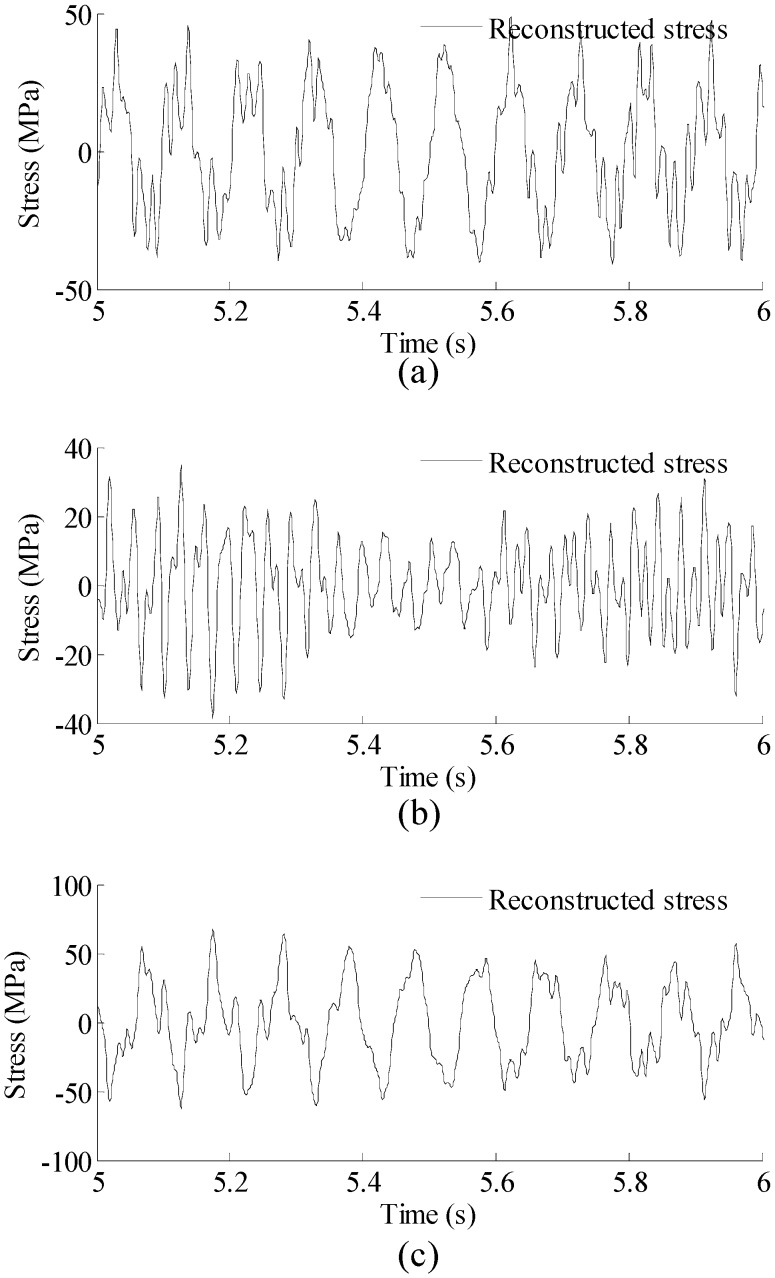
Reconstructed stress responses for three locations (Loc. 1–3) in Figure 2. Results are concentrated on 5–6 s for clear presentation. (**a**) Results for Loc. 1; (**b**) Results for Loc. 2; and (**c**) Results for Loc. 3.

Correlation coefficients between the reconstructed strain responses and the theoretical strain responses for the three locations are 0.981, 0.978, and 0.981, respectively, indicating that the proposed methodology produces reliable and accurate results over the entire structure.To reconstruct the stress responses, the reconstructed strain responses are used in Equation (14). The material matrix for this 1D beam problem is a 1 × 1 matrix (a scalar) of c=[E] in which *E* is the Young’s modulus of the material. The reconstructed stress results are shown in Figure 6.

The beam example demonstrates the overall reconstruction procedure and the effectiveness of the proposed method. Next, a numerical airfoil example is presented to investigate the performance of the method for complicated problems.

### 3.2. Example 2: A Simplified Airfoil Structure Model Example

**Figure 7:**
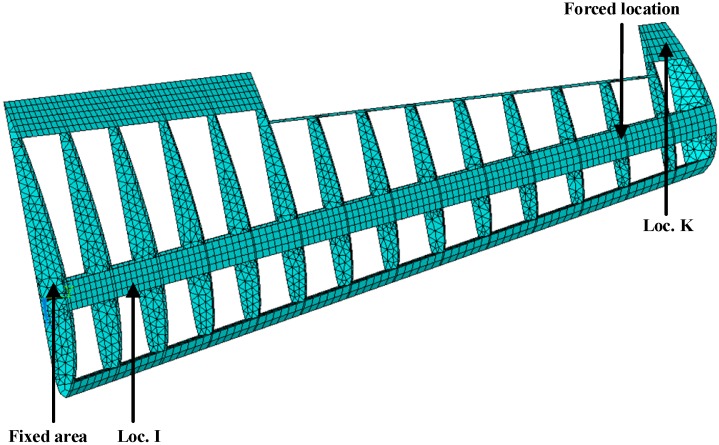
FE model of the simplified airfoil structure.

The proposed methodology can be directly applied to structural level reconstruction analysis. To demonstrate the basic idea, a 19956-DOF simplified airfoil structure model is used here. The finite element model diagram of the structure and its dimensions are shown in Figure 7. The structure is a simplified airfoil without skin and it has extents of 3.49 m, 0.11 m, and 0.95 m in x-axis direction, y-axis direction, and z-axis direction, respectively.

The element type is solid185 so that it is automatically meshed with hexahedron or (reduced) tetrahedron elements in ANSYS. Each of the elements has 8 nodes and 24 DOFs in total. Each node has 3 DOFs, namely the displacements of x, y, and z-axis directions. All the nodes attached to the airframe (i.e., *z* = 0) are prescribed and the structure has 19,956 DOFs. Required properties of the FE modeling in ANSYS are listed in Table 2.

Near the end of the airfoil (node 3224 in FE model), transient force with the amplitude of 100N in the y-axis direction is applied to simulate the ambient excitations for the first 0.01 s. The Loc. K (element 1847 in FE model) in Figure 7 is used as the location of the actual strain measurements. Sensor measurements are presented by transient dynamic analysis in ANSYS and 5% RMS noise terms are added to the deterministic results to represent the measurement uncertainty. The sampling frequency is 1000 Hz. The noise terms are generated using the same methods in the beam example. Without loss of generality, the Loc. I (element 1082 in FE model) in Figure 7 is arbitrarily chosen to represent the location of interest. Both the strain measurement location and the reconstructed location are shown in Figure 7.

#### 3.2.1. Strain and Stress Response Reconstruction

**Figure 8:**
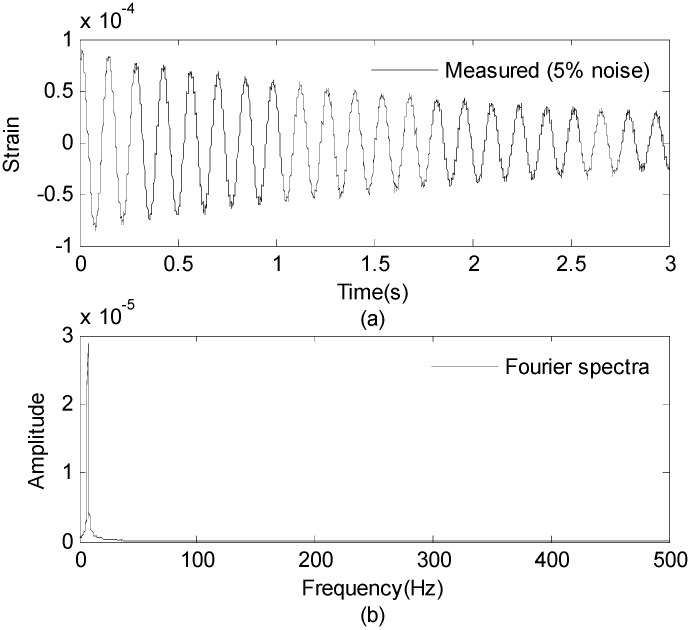
Strain measurement data and Fourier spectra of the data. (**a**) Strain measurement data (0–3 s); and (**b**) Fourier spectra of the measurement data (0–3 s).

**Figure 9:**
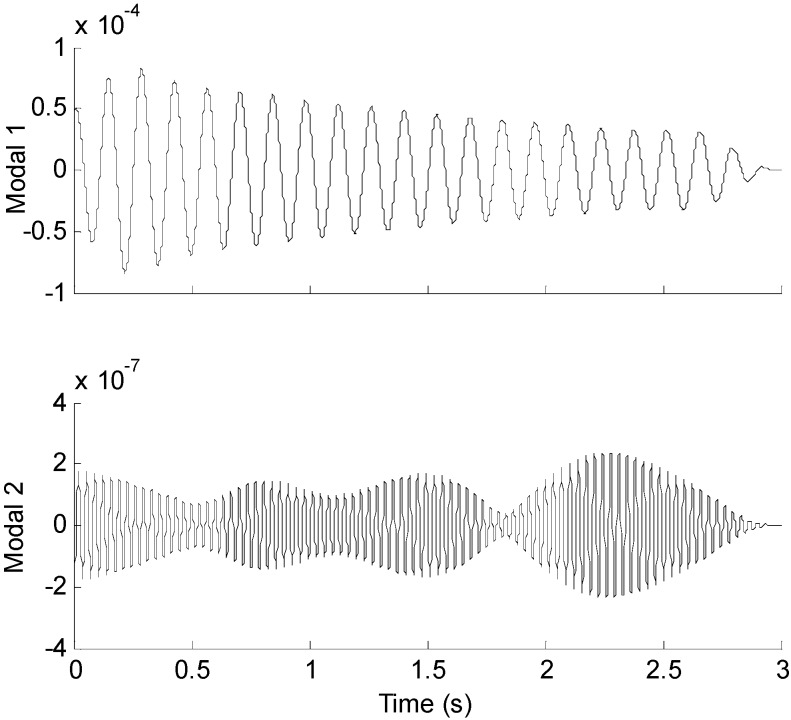
Modal responses of the strain measurement data obtained by EMD method with intermittency criteria.

**Figure 10:**
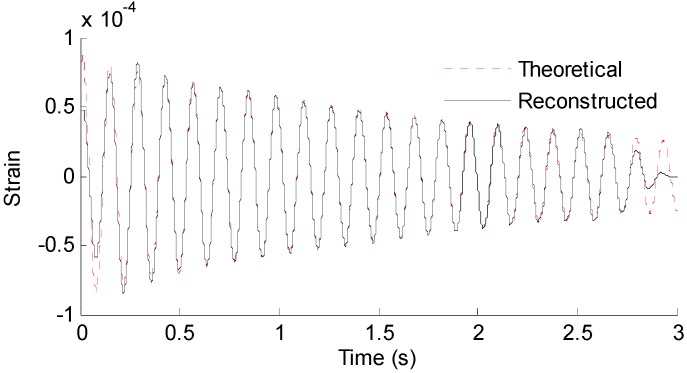
Reconstructed and theoretical strain responses for the location of interest shown in Figure 7.

**Figure 11:**
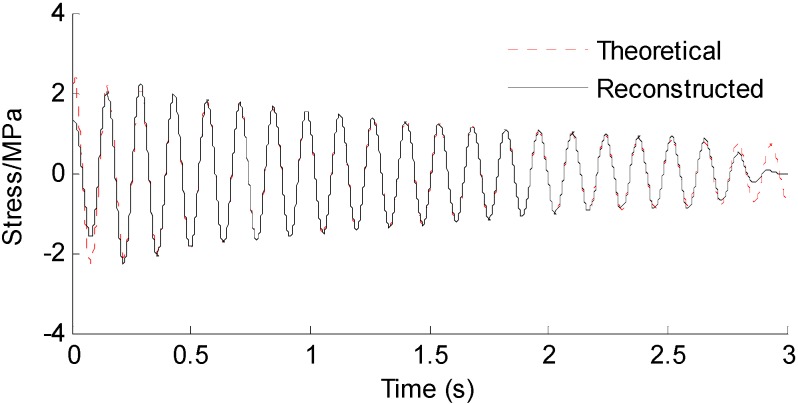
Reconstructed and theoretical bending stress responses for the Loc. I labeled in Figure 7.

Three seconds of synthesized strain measurement data with 5% RMS noise terms are presented in Figure 8a. The Fourier spectra of the strain measurement data indicates a significant frequency component as shown in Figure 8b. The frequency is used to design the band-pass filter for the modal responses extraction. Figure 9 presents the modal responses obtained by the EMD method with intermittency criteria. The reconstructed strain responses for the location of interest I, and the theoretical results are compared in Figure 10. The reconstructed stress responses based on the reconstructed strain results are shown in Figure 11. The correlation between the reconstructed strain responses and the theoretical strain results is 0.9849.

#### 3.2.2. Effect of Measurement Noise to Reconstructed Strain and Stress Responses

**Figure 12:**
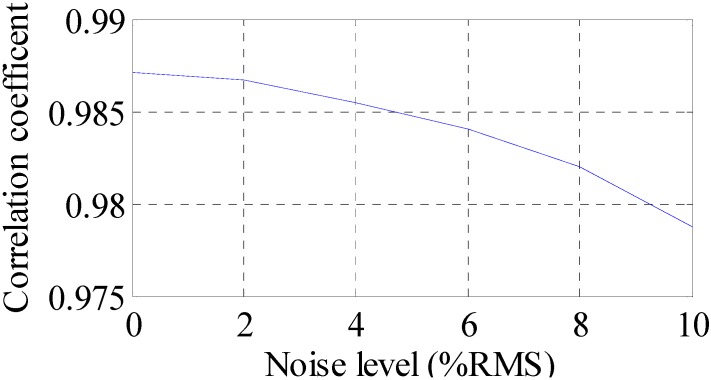
Reconstruction performance measured in correlation coefficient under different noise levels.

To investigate the effect of measurement noise to the reconstructed strain and stress responses, several numerical cases are studied. To represent realistic situations, the noise signals are Gaussian pulse processes with RMS setting to a percentage of the largest RMS of the calculated strain responses. The percentage value is defined as noise level. For example, a 5% noise level is to generate noise components from Gaussian pulse processes with RMS setting to 5% of the largest RMS of the strain responses. The noise components are added to the strain responses and the results are used as the representative noisy strain measurement data. Correlation coefficient is used as a metric to evaluate the similarity between the theoretical responses and the reconstructed responses for bending stresses. RMS is set to taking value from 0% to 10% with 1% increment. At each of the RMS settings, bending stress responses at Loc. I (as shown in Figure 7) are reconstructed based on the strain measurement at Loc. K (as shown in Figure 7). The correlation coefficient between the reconstructed stress responses at Loc. I and the theoretical stress responses (also with noise components) is calculated. Results for all RMS settings are presented in Figure 12.

It can be seen that the performance is degraded with the increase of noise level. The overall performance of the reconstruction method is larger than 97.5% when noise levels are not larger than 10% RMS in this example. Next, the experimental validation of the time domain strain and stress reconstruction method is presented.

## 4. Experimental Validation

### 4.1. Experimental Setup

**Figure 13:**
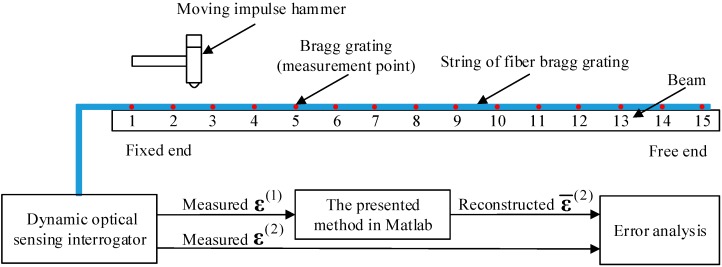
Schematic diagram of the experimental setup for the validation of the presented strain/stress reconstruction method.

The test specimen used in this study is a standard cantilever beam which is commonly used in air vehicle and spacecraft. Figure 13 depicts the experimental setup used in this work. As shown, the beam is set up as a cantilever supported by four threaded bolts at the fixed end and is set free at the other end. Optical fiber sensors are employed in this study to obtain strain measurements. One potential benefit to choose optical fiber is that the optical fiber is light and thin which can reduce the effects of extra mass.

**Figure 14:**
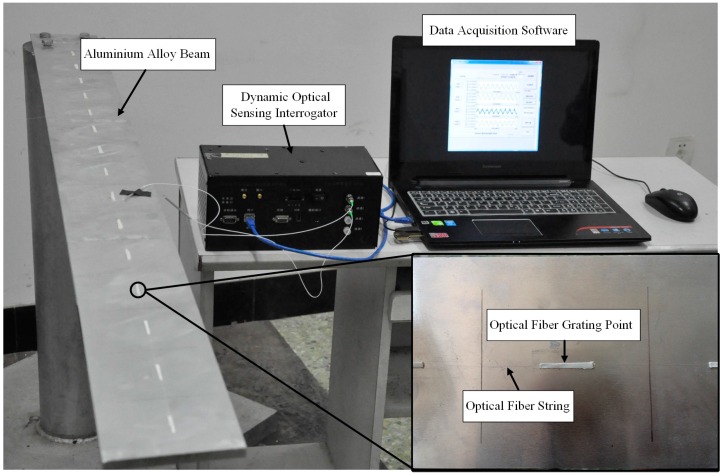
The entire experimental setup of the study.

To investigate the influence of the sensor location to the performance of the proposed time domain strain/stress reconstruction method, a total of 15 optical fiber strain sensors are bonded to the upper surface of the beam at the equivalent locations. The beam is divided into 15 equal portions for finite element simulation. The beam is excited with a moving impulse hammer to simulate the impulse force. Then the responses of the optical fiber sensors are simultaneously collected through the dynamic optical sensing interrogator (type: sm130, from Micron Optics Inc., Atlanta, GA, USA). The sampling frequency is kept at 2 kHz. Figure 14 presents the entire experimental setup of the study. The specifications of the beam are listed in Table 3.

### 4.2. Results and Discussion

**Figure 15:**
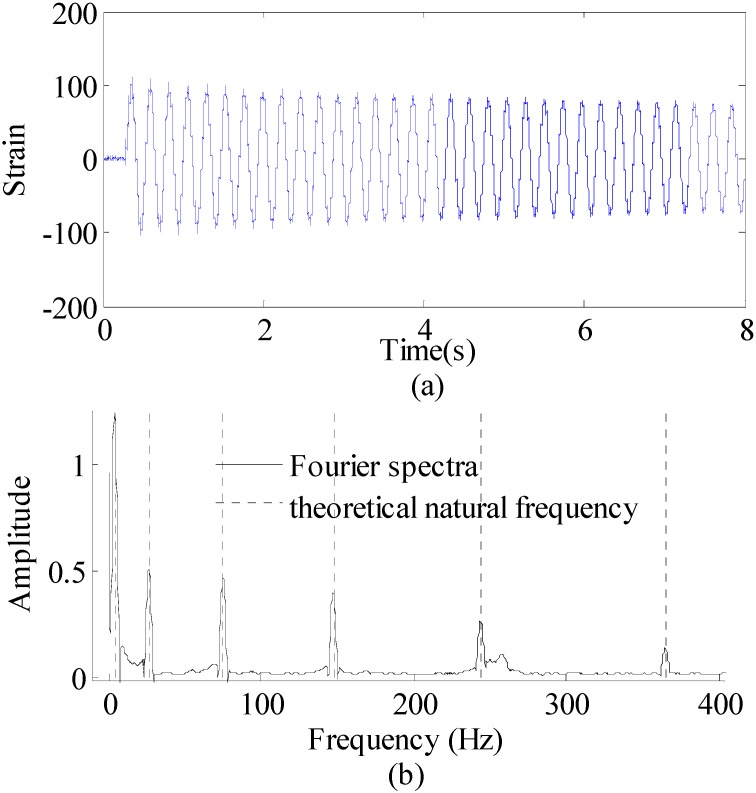
Strain sensor measurement data and Fourier spectra of the data. (**a**) strain sensor measurement data (0~8 s), and (**b**) Fourier spectra of the measurement data (0~8 s).

First, the proposed method is used to reconstruct the strain responses at unavailable location (15-th measurement point) from accessible location (1st measurement point). The measured strain data of the 1st measurement point in Figure 13 with the duration of the first 8 s are presented in Figure 15a. The unit of strain data is με. The Fourier spectra of the optical fiber measurement data indicate six significant frequency components as shown in Figure 15b. For the higher modes, it is difficult to detect them because the participation factors are relatively small. However, the higher model will have trial influence on reconstruction results due to the low participation factors. Only dominant modes are efficient for accurate reconstructions. This conclusion has been confirmed by later results. These six frequencies are used to design band-pass filters for the modal responses extraction in this case.

**Figure 16:**
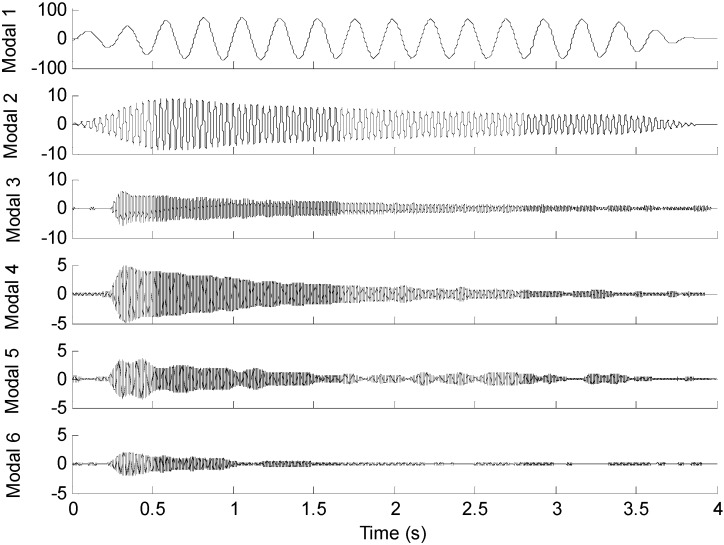
Modal responses of the 15-th optical fiber measurement data obtained by EMD method with intermittency criteria.

**Figure 17:**
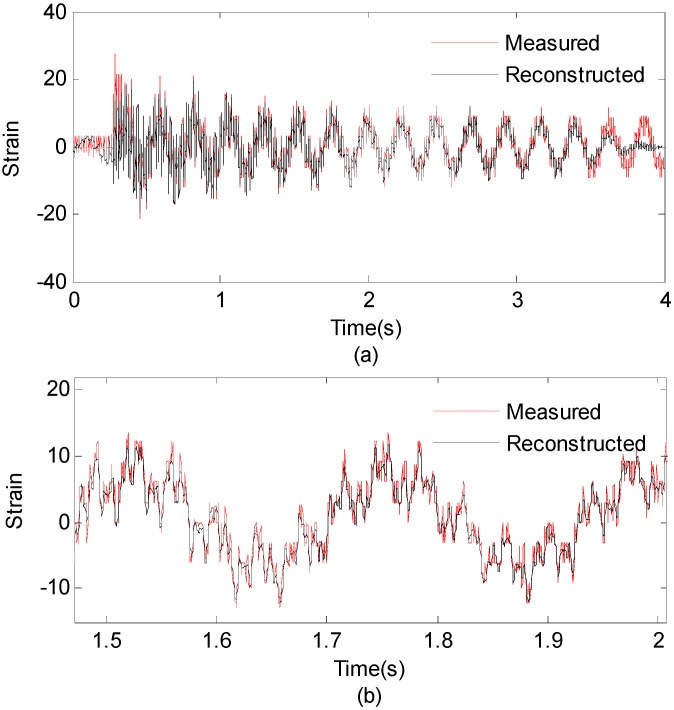
(**a**) Reconstructed and theoretical strain responses for the location of interest; (**b**) Results are concentrated on 4–6 s for clear presentation.

To reconstruct strain response at the 15-th measurement point, the EMD method with intermittency criteria is employed to obtain modal responses. Table 4 gives frequency ranges for each band-pass filter. Figure 16 presents the modal responses obtained by the EMD method with intermittency criteria. The reconstructed strain response at the 15-th measurement point and the theoretical result are compared in Figure 17a. Figure 17b is the partial time-history chart of Figure 17a from 1.5 to 2 s. It is observed from Figure 17 that the reconstructed responses agree well with the theoretical solution except for the boundary region. The reconstruction errors in the beginning and end of signals are caused by the Gibbs phenomenon which has also been referred as boundary/end effect in EMD method [14], which has been widely discussed as the intrinsic weakness of EMD. One of the possible solutions for this problem is using the mirroring method to extend signal envelopes from both ends, thus effectively eliminating end effects. The solution and verification of the Gibbs phenomena is also a worthy research in future work. The correlation between the reconstructed strain responses and the measured strain results is 0.9222. This case indicates that the proposed method is suitable for strain response reconstruction on practical complex structures. Following the basic case, effects of the sensor number, sensor location and mode number are studied as three cases in detail.

### Case 1. Effect of Sensor Number

**Figure 18:**
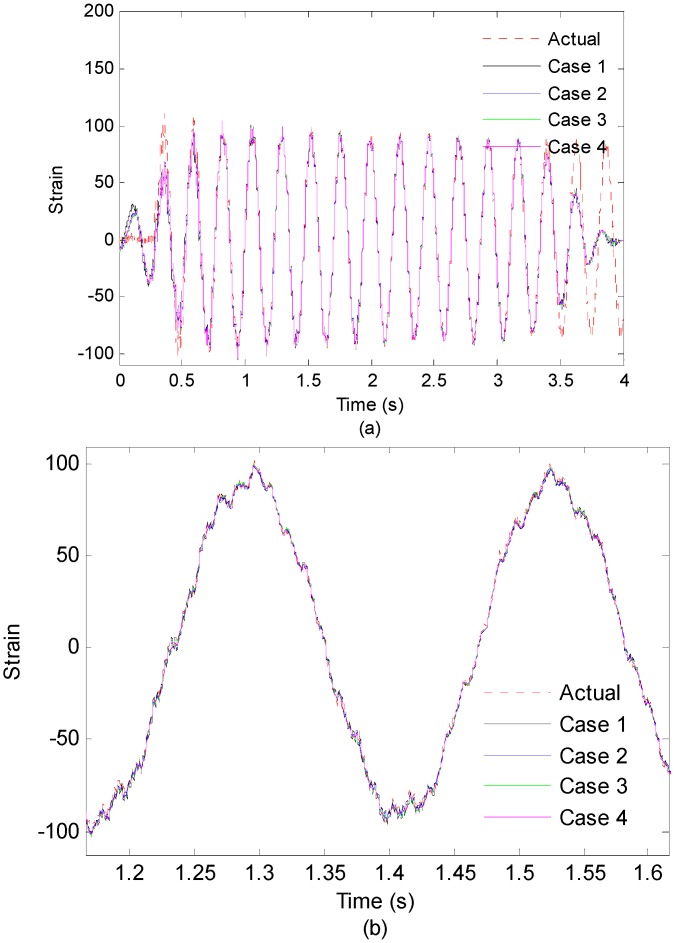
(**a**) Measured strain responses and reconstructed strain responses (using 1,2,3,14 measurement points) of the 5-th optical fiber measurement point on first 4 s; (**b**) Results are concentrated on 1.2–1.6 s for clear presentation.

In order to investigate the effect of the sensor number on the performance of the proposed strain/stress reconstruction method, 14 measurement points are taken into consideration and divided into four cases in the experiment. Table 5 presents the case set of measurement points for reconstruction and correlation coefficients calculated between measurement data and reconstruction data. In this example, the information from multiple measurement points are combined and used for reconstruction process, and 5-th measurement point is chosen to be the location of interest. In case 1, only the strain data of 7-th point is used to reconstruct strain responses of the location of interest. In case 2, two measured strain data set from 3-th and 12-th points are used to perform the strain reconstruction and the obtained reconstruction responses are averaged. In case 3 and case 4, three measured strain data set from 3-th, 7-th and 12-th points and 14 measured strain data set from the rest points (except 5-th) are used to perform the strain reconstruction, respectively. Similarly, the obtained reconstruction responses are averaged. Figure 18 shows measurement strain data and reconstructed strain responses of the 5-th optical fiber measurement point in the first 4 s.

From Figure 18, it is observed that increasing the number of the measurement points has trivial effects on the accuracy of the reconstruction results, which is consistent with the simulation results in [14]. However, in the real situation, increasing the number of measurement points can improve the robustness of the data acquisition in case of measurement points are broken or record incorrect data.

### Case 2. Effect of Sensor Location

**Figure 19:**
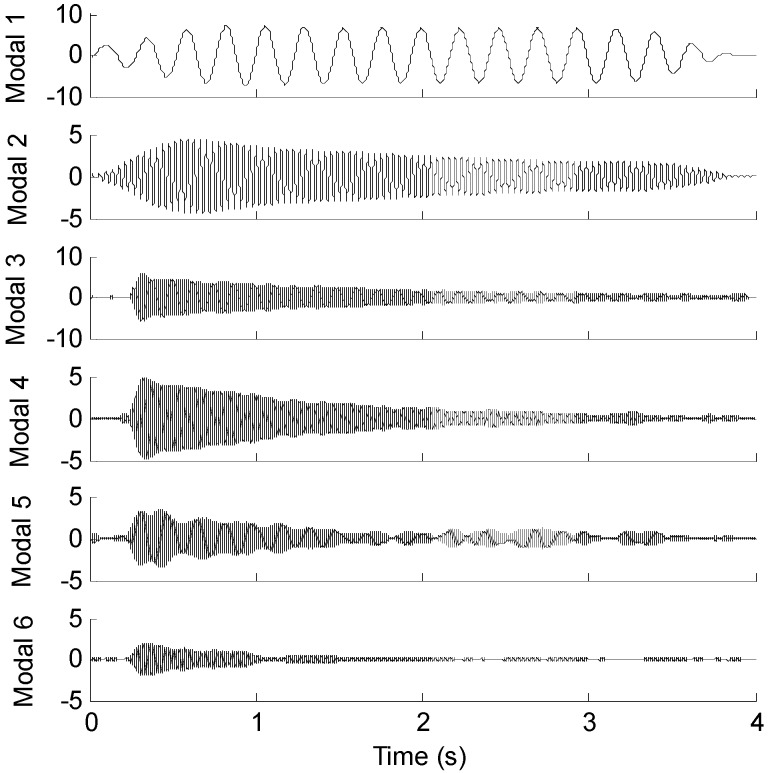
Modal strain responses of the 15-th optical fiber measurement data obtained by EMD method with intermittency criteria.

**Figure 20:**
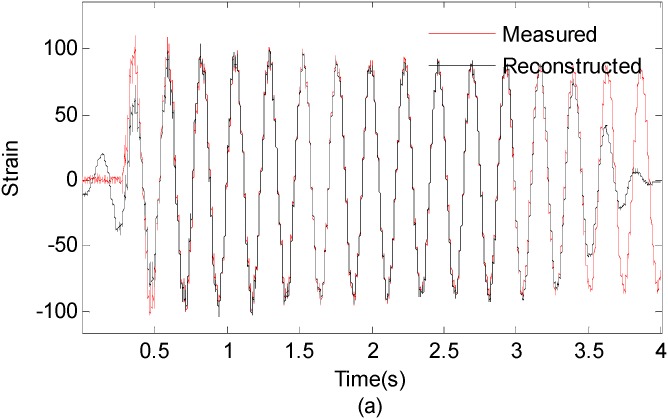

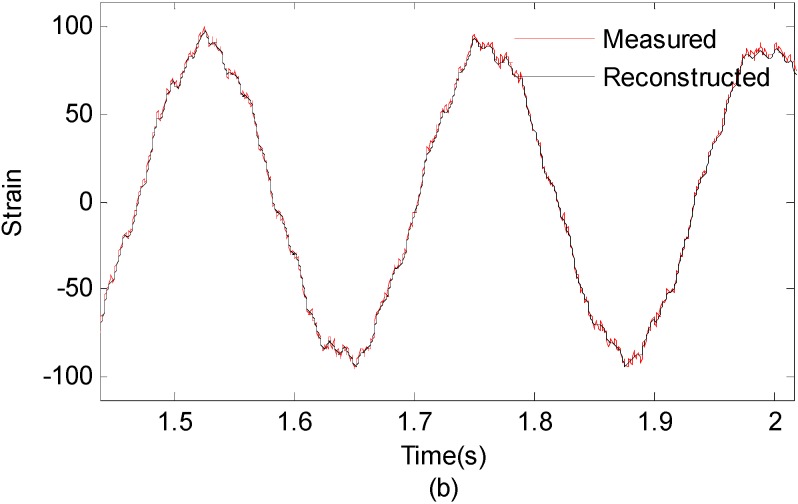
(**a**) Measured strain responses and reconstructed strain responses of the 1st optical fiber measurement data; (0~4 s) (**b**) Results are concentrated on 1.5~2 s for clear presentation.

In real situation, predicting the response of the fixed end of the beam from measurements taken near the free end may be more problematic. In order to investigate the effect of the sensor location on the proposed method in practical experiment, the measured strain signal collected from the free end (the 15-th measurement point) is used to reconstruct the strain responses near the fixed end (the 1st measurement point). Figure 19 presents the first six modal responses obtained by the EMD method with intermittency criteria. Figure 20a shows measured strain responses near the fixed end and reconstructed strain responses in the first 4s. Figure 20b shows measured strain responses and reconstructed strain responses in 1.5~2 s for clear presentation.

It is observed from Figure 20 that the reconstructed response agrees well with the theoretical solution. The correlation between measured strain responses near the fixed end and reconstructed responses results is 0.9522. This case study demonstrates the capability of the proposed method for handling different sensor location cases. However, clarifications are made as follows: Firstly, in this case study, signals from different locations have the same signal-to-noise ratios hypothetically. However, signal-to-noise ratio can be quite different for different locations due to the different signal amplitude in real engineering applications. The noise will dominant the signal for very small signal amplitude cases. Thus, the sensor location will have significant effect on the accuracy of reconstruction results under this circumstance. Secondly, if the sensor is installed at or near the nodal point of a particular mode, the strain responses may not capture all the excited modes. In such cases, an IMF for this specific mode will be close to zero. Therefore, it is difficult to reconstruct the strain responses at the point of interest.

### Case 3. Effect of Mode Number

**Figure 21:**
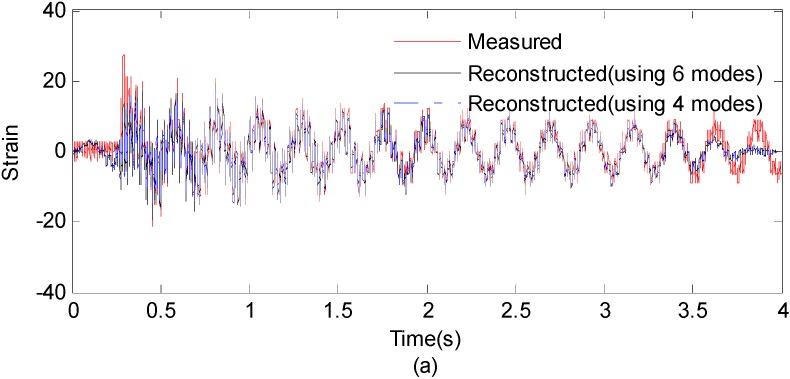

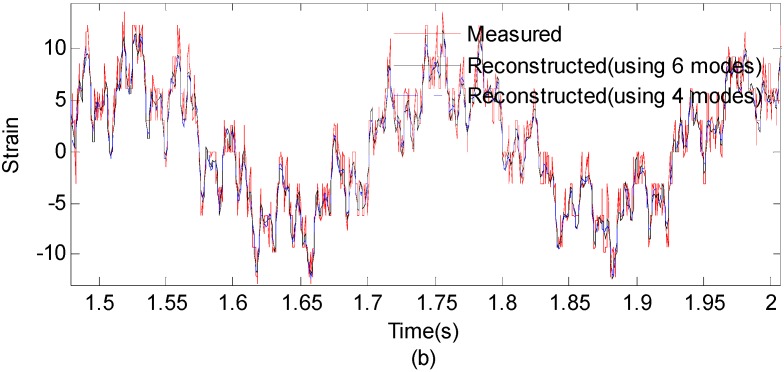
(**a**) Measured strain responses and reconstructed responses using six modes and four modes; (0~4 s) (**b**) Results are concentrated on 1.5~2 s for clear presentation.

Another case study is performed to further investigate the relationship of performance of the reconstruction procedure and the mode number. The strain responses at the 1st measurement point is used to reconstruct the strain responses at the 15-th measurement point. In the two situations, the first four and six modes are chosen to reconstruct the strain responses at the point of interest. Figure 21 presents the results for measured strain response and reconstructed responses using six modes and four modes, respectively.

The correlation between the reconstructed strain responses using four modes and the measured strain results is 0.9052, which is 0.9222 for the six modes situation. It can be seen from the comparison between the reconstruction results using four modes and results obtained using six modes that it produces little effect when the number of used modes raises. This is possible because the frequency components in the Fourier spectra with low amplitude have no significant effect on the performance of the proposed method. Therefore, the accuracy of reconstruction results is effected little when the number of used higher modes increases. In some time or cost constrained cases, the higher modes can be safely discarded.

## 5. Conclusions

The current study extends the methodology in previous work [14] to time domain strain/stress reconstruction approach using limited sensor data and implements four sets of experiments to validate the proposed work. Sparse and remote strain measurements from the existing structural health monitoring (SHM) system are directly used to reconstruct the strain and stress responses for critical spots without direct sensor measurements. EMD method with intermittency criteria is first employed in the proposed method to decouple the remote strain measurements into modal coordinates. Then the extrapolation of the strain and stress modal responses is made using the derived strain and stress transformation function. The overall method is demonstrated using a beam structure and a more complex 19,956-DOF simplified airfoil structure. Four sets of experiments conducted on an aluminium alloy cantilever beam are employed to investigate the effectiveness and accuracy of the proposed method. A total of 15 optical fiber sensors are bonded on the upper surface of the beam to collect strain data. Effects of sensor locations, the number of sensors and the number of modes are investigated in detail by experimental study.

However, we are aware of some limitations that should be stated as follows: firstly, the performance of the band-pass filter for separating different modes critically affects the accuracy of the reconstruction results. Therefore, the proposed method is not suitable for problems with equal or closely spaced modes. Second, the proposed method is suitable for stochastic excitations and transient excitation, which the external forces can be regarded as random, but it is not suitable for cases which the external force which has deterministic frequency, such as periodic excitation. In this situation, the frequency component from excitation forces which is not associated with any mode will be dominant and the natural mode components will not show in the Fourier spectra. 

Based on the current study, several conclusions can be drawn:

(1) In this study, a time domain strain/stress reconstruction method based on EMD is proposed. According to numerical analysis results, the proposed method can produce results which are very close to theoretical solutions considering a practical noisy measurement system. The reconstructed results have an overall correlation coefficient larger than 0.975 under 10% RMS noise settings. The discrepancy between actual measurements and reconstruction results at the boundary region are possibly caused by the end boundary effect of EMD method.
(2) Four sets of experiments, associated with basic example, sensor number, sensor location and mode number, verified the effectiveness of the time domain strain/stress reconstruction method in successfully reconstructing the strain response in location of interested. The results indicate that increasing the number of the measurement points has trivial effects on the reconstruction accuracy under ideal experimental circumstance. However, increasing the number of the measurement points may decrease the uncertainty (imposed by measurement noise or mishandling) for real engineering applications. Thus, more sensor measurements will commonly lead to higher reconstruction accuracy.
(3) For the sensor location, two specific sensor locations should be avoided for reliable strain/stress response reconstruction: (a) locations where have low signal-to-noise ratio. In such case, the measured strain data are corrupted by noise, which will lead to inaccurate reconstruction results; (b) locations at or near the nodal points. In such locations, it may not capture all excited modes of the strain responses.
(4) For the mode number, the higher modes will have little influence on the accuracy of the reconstruction, because of their low participation factors in Fourier spectra. Only dominant modes are efficient for accurate reconstructions.

Future works will focus on applying this method on the structures with damage and composite materials. And the solution and verification of the Gibbs phenomena is also undergoing.

## Figures and Tables

**Table 1:** Frequency ranges for each band-pass filters for the beam problem.

Mode	1	2	3	4
Identified frequency	10.38	28.69	56.22	93.15
Passband corner frequency (Hz)	[8–9]	[22–26]	[46–52]	[80–87]
Stopband corner frequency (Hz)	[11.5–13]	[31–36]	[61–67]	[98–105]

**Table 2:** Properties of FE model in ANSYS.

Property	Value
Material	Aluminum 7075
Element type	Solid185
Young’s modulus *E* (GPa)	72
Poisson’s ratio *ν*	0.33
Mass per unit volume *ρ* (kg/m^3^)	2.81 × 103
Number of elements	14,951

**Table 3:** Dimensions and properties of the aluminium alloy beam.

Property	Value
Material	Aluminum 7050
Length	1.36 m
Width	0.12 m
Thick	0.01 m
Young’s modulus *E* (GPa)	7.17
Poisson’s ratio *ν*	0.33
Mass per unit volume *ρ* (kg/m^3^)	2.81 × 103

**Table 4:** Frequency ranges for each band-pass filters for the experiment.

Mode	1	2	3	4	5	6
Identified frequency	4.39	27.23	75.69	148.22	244.15	365.26
Passband corner frequency (Hz)	[3–4]	[25–26]	[65–70]	[135–140]	[226–231]	[350–355]
Stopband corner frequency (Hz)	[5–6]	[27.5–28.5]	[80–85]	[160–165]	[257–262]	[375–380]

**Table 5:** Experimental results for 4 cases of the sensor number example.

	Case 1	Case 2	Case 3	Case 4
Measurement points for reconstruction	7-th	3-th, 12-th	3-th, 7-th, 12-th	the rest points except 5-th
Correlation coefficient	0.9497	0.9516	0.9519	0.9519
